# Identification of a Feline Panleukopenia Virus from Captive Giant Pandas (*Ailuropoda melanoleuca*) and Its Phylogenetic Analysis

**DOI:** 10.1155/2023/7721487

**Published:** 2023-05-19

**Authors:** Yuqing Yang, Yi Geng, Ping Ouyang, Yunli Li, Hongrui Guo, Huidan Deng, Rong Hou, Weimin Lai, Dongsheng Zhang, Songrui Liu

**Affiliations:** ^1^College of Veterinary Medicine, Sichuan Agricultural University, Wenjiang 611130, Sichuan, China; ^2^Chengdu Research Base of Giant Panda Breeding, Sichuan Key Laboratory of Conservation Biology for Endangered Wildlife, Chengdu 610081, Sichuan, China

## Abstract

The host range of feline panleukopenia virus (FPV) is expanding and is a serious threat to both captive and free-range endangered wildlife. The FPV named FPV-am2020 was isolated from fecal samples from four diarrheal captive giant pandas in 2020, and pathogenicity and phylogenetic analysis were conducted in this study. Three-month-old cats challenged with FPV-am2020 experienced 100% mortality. The complete FPV-am2020 sequence was determined and comprised 5277 base pairs (bp), 36.76% GC content, and two open reading frames. According to the phylogenetic analysis of whole genome sequences and *VP2* gene sequences, FPV-am2020 was closely related with MG764511.1 (isolated from captive lions in China, 2015), KX685354.1 (isolated from captive tigers in China, 2016), and KX900570.1 (isolated from captive jaguar in China, 1986). Furthermore, the study identified a G299E mutation in *VP2* which was a key residue involved in phenotype changes in FPV. Thus, increased surveillance of FPV mutant isolates must be enacted to protect giant pandas against potential viral threats.

## 1. Introduction

Feline panleukopenia virus (FPV), canine parvovirus-2 (CPV-2), mink enteritis virus, and raccoon parvovirus are all members of the carnivore protoparvovirus 1 (CPPV-1) species (genus *Protoparvovirus*, subfamily *Parvovirinae*, family *Parvoviridae*) [1]. FPV has two open reading frames encoding the nonstructural proteins *NS1* and *NS2* and the capsid proteins *VP1* and *VP2*. Several amino acid residues at *VP2* gene influence antigenicity and the host ranges [1]. Residue 299 is involved in CPV-2 infection by binding to the transferrin receptor (TfR). Many carnivores are susceptible to FPV and include banded linsang, mongoose, red fox, otter, snow leopard, serval, Siberian tiger, binturong, raccoon, coati, ringtailed cat, and mink [2–5]. Of these report cases, cats when challenged with FPV from the Siberian tiger developed hemorrhagic enteritis, dilated crypt lumina, and collapsed villi [5]. More importantly, snow leopards infected with FPV developed hemorrhagic enteritis and/or fibrinous enteritis and died [6].

The giant panda (*Ailuropoda melanoleuca*) is a unique ursid listed as “vulnerable” by the International Union for Conservation of Nature and holds national treasure status in China. Viral diseases are common causes of death in giant pandas and a threat to both the *in situ* and *ex situ* conservation of the species. These viruses include canine distemper virus (CDV) [7], canine coronavirus (CCV) [8], rotavirus (RV) [9], and CPV-2. CPV-2 has also been detected in captive giant pandas and wild giant pandas recently rescued by serological surveys [10]. Recently, a new CPV-2a with a Q370R substitution in *VP2* was identified in giant pandas within China [11]. Moreover, several captive giant pandas have died from CDV infection [12], demonstrating the risk viral outbreaks pose to the *ex situ* conservation of the species. A giant panda in Sichuan, China, suffered diarrhea and died in February 2012; parvovirus was isolated from this individual and confirmed to have the characteristics of CPV-2 and FPV recombinant virus, and *VP2* gene analysis showed that the isolate was close to a monkey-associated FPV isolate [13, 14]. In 2020, four more captive giant pandas in Sichuan developed diarrhea and were diagnosed with FPV (FPV-am2020). In this study, we describe the isolation and identification of this FPV (FPV-am2020), the phylogenetic relationship, and pathogenicity in cats.

## 2. Materials and Methods

### 2.1. Samples and PCR Detection

Two captive giant pandas (one female cub and one male subadult) developed diarrhea and vomiting, and other two (adult males) had mild diarrhea and suffered from low spirits and decreased appetite at the Chengdu Research Base of Giant Panda Breeding (CRBGPB) in Sichuan in 2020. Fecal samples were collected from the abovementioned four giant pandas, which were all detected for CDV, CCV, and RV negatively by CDV Ag TEST (Rapigen, South Korea), CCV Ag TEST (Rapigen, South Korea), and Diagnostic Kit for RV (Lanzhou Institute of Biological Products Co. Ltd, China), respectively. The samples were dissolved in phosphate-buffered saline (PBS) (HyClone, USA), homogenized, centrifuged at 4700×*g* for 20 min at 4°C, and freeze-thawed three times. Then, the supernatant was treated with chloroform, filtered through 0.22 *μ*m membranes, and stored at −80°C. Total DNA was extracted from filtered supernatant using a DNA extraction kit (Tiangen Biotech, Co. Ltd., China) according to manufacturer's protocols. FPV was detected by PCR using the *VP2* (*VP2*-F: GGATGGGTGGAAATCACAGC,*VP2*-R: ATAACCAACCTCAGCTGGTC, product size: 845 bp) specific primers [6]. The PCR thermal cycling conditions were 3 min at 95°C, followed by 30 cycles of 15 s at 94°C, 15 s at 55°C, and 15 s at 72°C. The products were sequenced by Sangon Biotech Co. Ltd. and aligned using the BLAST (https://www.ncbi.nlm.nih.gov/blast).

### 2.2. Virus Isolation and Hemagglutination Assay

1.0 mL of supernatant was added into the feline F81 cell (National Collection of Authenticated Cell Cultures) monolayer in 20% penicillin-streptomycin (Solarbio, China). After incubation at 37°C for 1 h, an inoculum was removed and replaced with fresh Roswell Park Memorial Institute's 1640 medium (RPMI 1640) (HyClone, USA) supplemented with 5% fetal bovine serum (FBS, Sangon Biotech Co. Ltd, China) and 1% penicillin-streptomycin. Cells were incubated at 37°C for 7 days and observed every 6 h for cytopathic effects. When cytopathic effects were observed, cultures were harvested by freeze-thawing three times and centrifuging at 6100×*g* for 30 min at 4°C. Several twofold dilutions of viral supernatants (10^−2.8^ TCID_50_/100 *μ*L) were made in PBS (pH 5.7–7.2), commencing from a 1 ∶ 2 dilution. Then, 25 *μ*L of a suspension containing 1% pig erythrocytes (Hongquan Biotechnology Co. Ltd., China) and 0.5% bovine serum albumin (Biyuntian, China) were added to dilutions, and detected the results after 1 h at 4°C and expressed as the reciprocal of the highest sample dilution producing hemagglutination.

### 2.3. Pathogenicity Studies in Cats

Three-month-old healthy cats (*n* = 6), weighing 1078 g–1267 g housed at Sichuan Agricultural University were randomly divided into two groups: control (*n* = 3) and test group (*n* = 3). None of the cats in this study was previously vaccinated, and all cats were negative for FPV, feline calicivirus (FCV), and feline herpes virus (FHV) as determined through PCR, FCV Ag test (Rapigen, South Korea), and FHV Ag test (Rapigen, South Korea) screening, respectively. Stringent measures were adopted to prevent contamination during these studies. Cats were challenged with 1.0 mL FPV-am2020 (10^−2.8^ TCID_50_/100 *μ*L) via oral routes. Control animals were mock challenged with 1.0 mL RPMI 1640 supplemented with 5% FBS and 1% penicillin-streptomycin. Clinical symptoms, weights, and rectal temperatures were monitored, and rectal swabs were taken daily for PCR testing. After moribund, necropsies were performed and tissue samples including the heart, liver, spleen, lung, and kidney were collected for PCR testing and histopathology.

### 2.4. Microscopy

#### 2.4.1. Immunofluorescence Assay

Cells were fixed in 4% paraformaldehyde (Biyuntian) for 30 min. Cell membranes were permeabilized in Triton X-100 (Biyuntian) for 30 min at room temperature and then blocked for 30 min. Then, cells were incubated overnight at 4°C with an anti-FPV monoclonal antibody (Shenzhen Anti Biological Technology Co. Ltd., China) and incubated with a fluorescein isothiocyanate-conjugated sheep anti-mouse secondary antibody (Shenzhen Anti Biological Technology Co. Ltd., China) at room temperature in the dark for 2 h. Then, 4′, 6-diamidino-2-phenylindole (DAPI) (Solarbio) was used to stain the nucleus.

#### 2.4.2. Histopathology

Samples for histopathology were fixed in 10% neutral buffered formalin and processed using standard paraffin wax techniques. Sections (4 *μ*m) were stained in hematoxylin and eosin (H&E).

#### 2.4.3. Transmission Electron Microscopy (TEM)

F81 cells infected with FPV (72 h) were fixed in 3% glutaraldehyde in PBS at 4°C for 24 h. After postfixing in 1% osmium tetroxide, specimens were dehydrated in a graded acetone series and embedded in epoxy resin. Blocks were sectioned at 50 nm, stained in uranyl acetate and lead citrate, and TEM (JEOL, JEM-1400 FLASH, Japan) used to observe ultrastructural cell injuries induced by virus particles. Virus samples were also purified by ultracentrifugation, collected in PBS, and negatively stained in 2% phosphotungstic acid to observe virus particles under TEM.

### 2.5. Genome Sequencing and Phylogenetic Analysis

The FPV-am2020 genome was sequenced using Illumina NovaSeq technology (Illumina, NovaSeq 6000, USA). The DNA fragments were end-repaired to construct a DNA library. Raw sequencing data were filtered, and host contamination was removed. After assembly using BLASTN (version 2.9.0+), sequences were uploaded to the National Center for Biotechnology Information (NCBI) (https://www.ncbi.nlm.nih.gov/). The secondary structures of genome terminal structures were predicted using DNAMAN software (version 6, Lynnon Biosoft, Montreal, QC, Canada). The phylogenetic analysis between the whole genome sequences of CPPV-1 (FPV-am2020 and other 35 CPPV-1) and *VP2* gene sequences (FPV-am2020 and other 234 CPPV-1) was conducted. A total of 35 whole genome sequences and 234 *VP2* gene sequences of CPPV-1 were downloaded from the NCBI database and were aligned in MEGA7. Based on the index of substitution saturation (Iss) calculated in DAMBE 7.3.11, the data were not saturated [15]. The best substitution models as well as maximum likelihood (ML) trees were then evaluated with the IQ-TREE (version 1.6.12). A total of 1000 bootstrap replicates were analyzed to obtain nodal support values [16]. CPV-2 was used as the outgroup taxa, and then all the ML trees were visualized and exported with iTOL (https://itol.embl.de/) [17].

### 2.6. Analysis of Nucleotide Substitution and Nonsynonymous Substitution

The study made comparison of nucleotide substitution in *NS1* gene and nonsynonymous substitution in *NS1* protein between FPV-am2020 and other closely related CPPV-1 (MZ357122.1, MG764511.1, KX685354.1, and KX900570.1). Besides, we made comparison of nucleotide substitution in *VP2* gene and nonsynonymous substitution in *VP2* protein between FPV-am2020 and other closely related CPPV-1 (MZ357122.1, EU498680.1, EU498681.1, MG764511.1, KX685354.1, and KX900570.1). FPV (B strain) *VP2* protein (PDB ID: 1FPV) was selected from the PDB database (https://www.rcsb.org/). The similarity of *VP2* protein between 1FPV and FPV-am2020 was 99.14%. The spatial structure changes of *VP2* protein at 299 amino acid residues was constructed by the PyMOL Molecular Graphics System (Version 2.0, Schrödinger, LLC) using 1FPV model. The surface distribution of electrostatic potential energy was compared with Vacuum Electrostatics by PyMOL.

## 3. Results

### 3.1. Virus Identification and Pathogenicity

**Figure 1:**
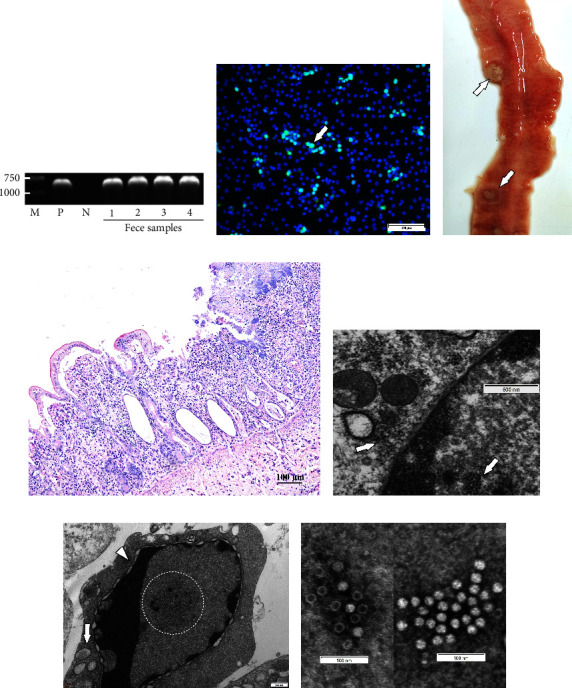
Virus isolation and identification. (a) The PCR results of *VP2* from the four giant pandas' feces. (b) FPV antigen was rendered fluorescent with FITC. The image was the merged image of FITC and DAPI (arrows). (c) Necrosis of Payer patches in the intestine of the infected cat in pathogenicity studies. The arrow indicated the necrosis area. (d) Villous atrophy, shed mucosal epithelium, necrotic crypt epithelial, and local inflammatory cell infiltration in the lamina propria. (e) The virus (arrows) in F81 cells by TEM. (f) The virus in the nucleus and apoptosis. The circle indicated virus, the arrow indicated the mitochondrial enlargement, and the arrowhead indicated the nuclear membrane swollen. (g) The virus particles by negatively stained.

FPV were positively detected in the feces of four giant panda based on the *VP2* PCR results (Figure 1(a)). From cell culture, typical cytopathic effects were observed in F81 cells (Figure S1A), and the FPV antigen was confirmed in F81 cells using indirect immunofluorescence (Figure 1(b)). The isolate was preliminarily termed FPV-am2020 (from the subadult male giant panda), which had the highest hemagglutination titer of 1 : 64 (pH = 5.9) (Figure S1B). The pathogenicity of FPV-am2020 was further evidenced through the artificial infection of cat, which resulted in the series of clinical symptoms such as diarrhea, biphasic fever vomiting, and anorexia and ultimately death. After dissection, the infected cat showed necrotizing inflammation of the intestines (Figures 1(c) and 1(d)) and stomach (Figure S2A), besides the infected cat also showed hemorrhagic enteritis (Figure S2B). Decreased number of lymphocytes and hyperplasia of reticuloendothelial cells were observed in the white pulp of the spleen (Figure S2C). Necrotizing inflammation could be seen in myocardium (Figure S2E) Moreover, *VP2* were detected in F81 cell culture supernatants and cat samples and most were detected positively for *VP2* except the thymus (Figure S3; Table S1), which verified the pathogenicity of FPV-am2020 by Koch's rule. Furthermore, the spherical virions (approximately 20 nm) could be observed in the nucleus and cytoplasm in F81 cells using TEM (Figure 1(e)), which resulted in the cell appeared obvious cytoplasmic edge shift, and nuclear membrane and organelle enlargement (Figure 1(f)). The half-moon nuclei with condensed chromatin (Figure 1(f)) indicated the apoptosis. By negatively stained purified virus (after ultracentrifugation), the virions could be observed that were round, without envelope, with a diameter of about 20 nm (Figure 1(g)). Intact virions have low electron density. Incomplete virion capsids are hexagonal bright circles (Figure 1(g)).

### 3.2. Phylogenetic Analysis of FPV-am2020

**Figure 2:**
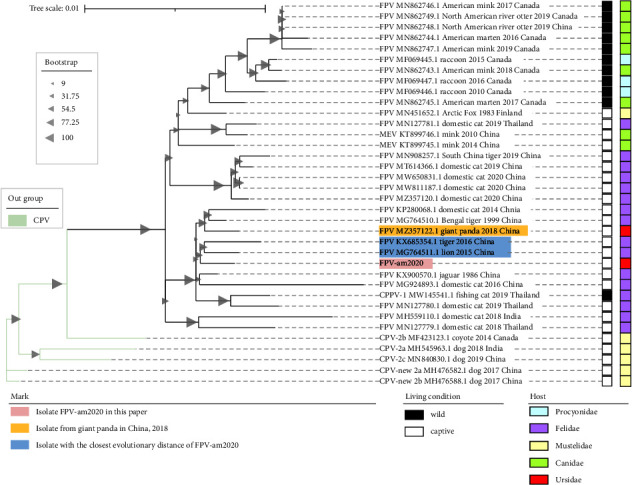
The phylogenetic analysis based on 36 whole genome sequences of CPPV-1. The best substitution model analyzed by IQ-TREE was HKY + F + I. The evolutionary distance was in the units of the number of base substitutions per site and the branch length scale bar indicates the evolutionary distance.

**Figure 3:**
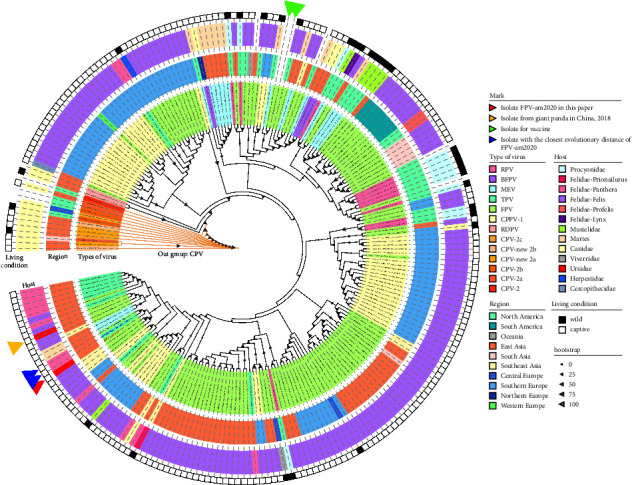
The phylogenetic analysis based on 235 *VP2* gene sequences of CPPV-1. The best substitution model analyzed by IQ-TREE was HKY + F + R2. Branch lengths were ignored.

After assembly, we generated the FPV-am2020 nucleotide sequence of 5277 bp, with a G + C content of 36.76% (GenBank: MZ712026). The sequence included the nonstructural (*NS1* and *NS2*) and capsid protein coding regions (*VP1* and *VP2*), which contained the palindromic sequence from the 3′ and 5′ end of the genome-assumed Y-shaped and U-shaped configurations, respectively (Figure S4).Based on 36 whole genome sequences of CPPV-1, we made the phylogenetic analysis using the maximum likelihood method by IQ-TREE (Figure 2; Table S2). The results showed that FPV-am2020 were most clustered with MG764511.1 (isolated from lion in China, 2015) and KX685354.1 (isolated from tiger in China, 2016), which also had a close distance to the genome FPV MZ357122.1 isolated from giant panda in 2018 (Figure 2). Moreover, due to the lack of genomic data, we collected the *VP2* sequences data of CPPV-1 to further investigate the relationship of FPV-am2020 (Table S3). Total 234 CPPV-1 strains were obtained and the phylogenetic analysis was performed using the maximum likelihood method by IQ-TREE. FPV-am2020 was classified in one cluster with the isolates of *Felis* and *Panthera* (Figure 3). The evolutionary tree showed that FPV-am2020 clustered with MG764511.1 and KX685354.1. However, FPV-am2020 was not close to the FPV vaccine (EU498680.1 and EU498681.1) in evolutionary distance (Figure 3).

### 3.3. Analysis of Nucleotide Substitution and Nonsynonymous Substitution

**Figure 4:**
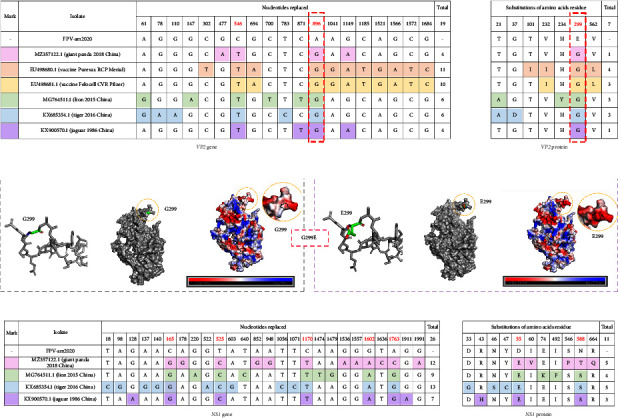
Nucleotide substitution in *NS1* gene and *VP2* gene and nonsynonymous substitution in *NS*1 protein and *VP2* protein. (a) *VP2* gene and *VP2* protein. Mutations are colored differently and important mutations are marked in red. (b) Spatial structure changes and surface distribution of electrostatic potential energy of G299E of *VP2* protein of 1FPV. (c) *NS1* gene and *NS*1 protein. Mutations are colored differently and important mutations are marked in red.

Finally, we evaluated the gene substitution of FPV-am2020 with several FPV strains that had most evolutionary relationship. Based on the analysis of *VP2* gene, FPV-am2020 had four nucleotide substitution compared with MZ357122.1, and eleven, ten, six, six, and four nucleotide substitution with EU498680.1, EU498681.1, MG764511.1, KX685354.1, and KX900570.1, respectively. These substitutions led to the nonsynonymous substitution in *VP2* protein, and the positions 896 led to nonsynonymous substitution of FPV-am2020 at residue 299 (Gly ⟶ Glu) (Figure 4(a)), which changed the spatial structure of the protein. Fifteen kinds of rotational isomers were formed, among which the rotational isomer with the least steric hindrance was formed and the strain equaled 23.49. The mutation also changed surface distribution of electrostatic potential energy. The negative potential energy value increased significantly, and the molecular surface became negatively charged (Figure 4(b)). Besides, through the analysis of *NS1* gene, FPV-am2020 had twelve nucleotide substitution compared with MZ357122.1, and nine, thirteen, and seven nucleotide substitution with MG764511.1, KX685354.1, and KX900570.1, respectively. Among them, positions 165 and 1763 led to nonsynonymous substitution at residue 55 and 588 of protein, respectively (Figure 4(c)).

## 4. Discussion

The FPV host-range is expanding (Figure 3) and is a serious threat to captive and free-range endangered animals due to its high mortality and transmission rates [18, 19]. In this study, FPV-am2020 was isolated and identified from captive giant pandas. As a result of the giant panda as a conservation of wild animals, cats were chosen to participate in the artificial infection challenged with FPV-am2020, which developed diarrhea consistent with the diseased giant pandas and showed a 100% mortality. In 2006, fatal infection caused by FPV occurred in a white tiger and an African lion in a zoo in Portugal [19]. Necropsy of the tiger and lion revealed catharral enteritis and severe hemorrhagic enteritis, respectively [19]. In addition, the histopathologic examination showed extensive necrosis of the crypts and the villi [19]. Thus, the virus could be a threat to giant pandas, especially the young cubs due to their under developed immune system. Under the potential threat of FPV, it is important to trace the origin of the FPV-am2020 for the protection of giant pandas.

According to the phylogenetic analysis of the whole genome sequences and *VP2* gene sequences, FPV-am2020 was closely related with four isolates that all isolated from captive animals. The captive animals are kept in close confines; after the introduction of the virus, it can spread easily, causing rapid infection. It was speculated that the stray cats were the source of the virus and it spread amongst the giant panda populations. This transmission mode was previously reported from snow leopards and servals in Sapporo Maruyama Zoo [5], where animals became infected with the virus without direct contact. In these cases, FPV was most likely spread through contamination of the surrounding environment [5]. Thus, the transmission source may be feral cats, or pet owners inadvertently carrying the virus on contaminated clothing. Therefore, strict biosecurity measures should be taken prevent the entry of feral cats to wild animal facilities as well as ensuring both animal keepers and the general public do not pose a contamination risk to captive wildlife.

Parvoviruses can cross species barriers to infect less susceptible hosts using single or a few mutations [20]. Several amino acid residue changes in the threefold spike of *VP2* could influence antigenicity and host range [21, 22]. Moreover, position 300 and adjacent residues at 299 and 301 in *VP2* were separately involved in adapting CPV-2 binding to the canine transferrin receptor; a change in CPV-2*VP2* residue 299 (Gly ⟶ Glu) caused the virus to lose its binding ability to canine TfR and 299-Glu severely restricts infection of CPV-2 in dog cells [23–25]. Although 299-Glu has not been observed in field isolates, it readily appears during *in vitro* passaging of cat cells infected with CPV-2, thus, it may arise as a compensatory mutation [23]. Newly emerging viruses acquire adaptive mutations to rapidly adapt to different host receptors and to facilitate transmission in new host species [26]. Metagenomic analyses showed that FPV was detected in giant panda feces in 2018 (MZ357122.1) and had a 299-Gly. The G896A (causing G299E amino acid mutation) point mutation in *VP2* was detected in both feces of giant pandas and F81 cells infected with FPV-am2020. This suggested that the mutation was stable and was derived from the natural infection of giant pandas and was not from the *in vitro* passaging of F81 cells. Such stable mutations may facilitate the spread of the virus in the host. However, it is not clear whether the mutations enhance the pathogenicity to the host. The interaction between mutations and TfR of giant panda in giant pandas remains to be studied. Moreover, the two isolated FPV isolates from giant pandas (MZ357122.1 and FPV-am2020) are not close to the FPV vaccine, and FPV-am2020 has the G299E amino acid mutation in *VP2* protein. Thus, the results suggested that the efficacy of commercial vaccines used with giant pandas require further investigation.

## Data Availability

The original contributions presented in the study are included within the article/Supplementary Materials; further inquiries can be directed to the corresponding author.

## Supplementary Materials

Supplementary Materials Figure S1: virus isolation and identification. (A) Cytopathic effects in F81 cells. (B) Relationship between pH of buffered solution and HA titer. Figure S2: the necropsy and histopathology of the challenged cats. (A) Necrosis in stomach. (B) Hemorrhagic enteritis. (C) Reduced lymphocytes in the white pulp. (D) Marked congestion in the lung. (E) Degeneration and necrosis of the myocardium. (F) Congestion. Figure S3: the PCR results. (A) The PCR results of cell culture supernatant. Lane 6 was cells control group and lanes 1–5 were positive samples of F81 inoculated with virus and different blindly passages. (B) The PCR results of the cat feces. (C) The PCR. Figure S4: the palindromic sequence from 3′ and 5′ end of the genome-assumed Y-shaped and U-shaped configurations, respectively. Table S1: the results of daily PCR swab testing and PCR on tissues after necropsy. Table S2: all sequences (accession numbers and data) used in the phylogenetic analysis based on 36 whole genome sequences of CPPV-1. Table S3: all sequences (accession numbers and data) used in the phylogenetic analysis based on 235 VP2 gene sequences of CPPV-1.
