# Supplementary material for: Identification of a Feline Panleukopenia Virus from Captive Giant Pandas (Ailuropoda melanoleuca) and Its Phylogenetic Analysis
Source: Transbound Emerg Dis. 2023 May 19;2023:7721487. doi: 10.1155/2023/7721487 (PMC12016729; doi:10.1155/2023/7721487)
Supplement: Supplementary Materials — Figure S1: virus isolation and identification. (A) Cytopathic effects in F81 cells. (B) Relationship between pH of buffered solution and HA titer. Figure S2: the necropsy and histopathology of the challenged cats. (A) Necrosis in stomach. (B) Hemorrhagic enteritis. (C) Reduced lymphocytes in the white pulp. (D) Marked congestion in the lung. (E) Degeneration and necrosis of the myocardium. (F) Congestion. Figure S3: the PCR results. (A) The PCR results of cell culture supernatant. Lane 6 was cells control group and lanes 1–5 were positive samples of F81 inoculated with virus and different blindly passages. (B) The PCR results of the cat feces. (C) The PCR. Figure S4: the palindromic sequence from 3′ and 5′ end of the genome-assumed Y-shaped and U-shaped configurations, respectively. Table S1: the results of daily PCR swab testing and PCR on tissues after necropsy. Table S2: all sequences (accession numbers and data) used in the phylogenetic analysis based on 36 whole genome sequences of CPPV-1. Table S3: all sequences (accession numbers and data) used in the phylogenetic analysis based on 235 VP2 gene sequences of CPPV-1. [file 7721487.f1.docx]

**Supplementary Material**


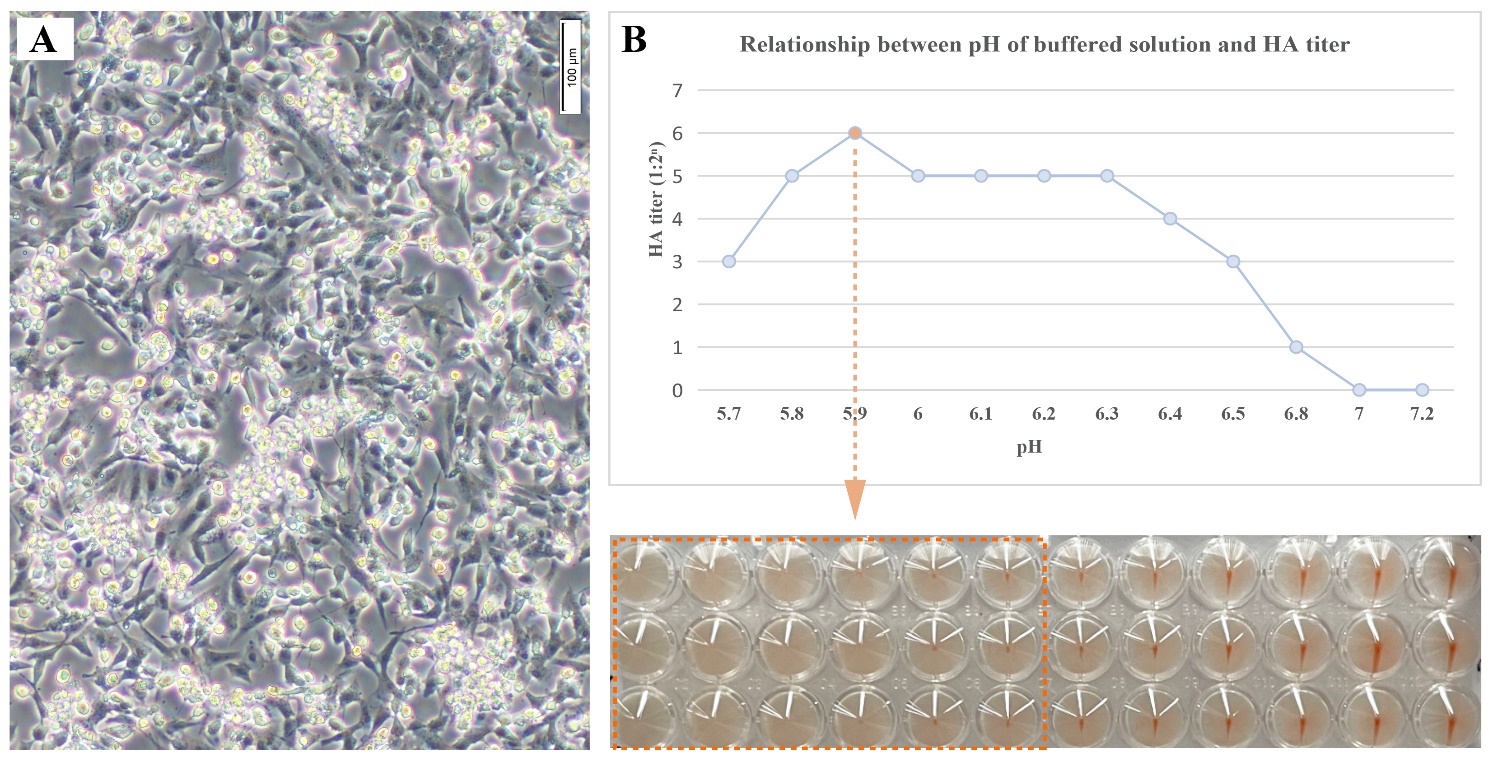


**Figure S1** Virus isolation and identification. (A) cytopathic effects in F81 cells. (B) Relationship between pH of buffered solution and HA titer.


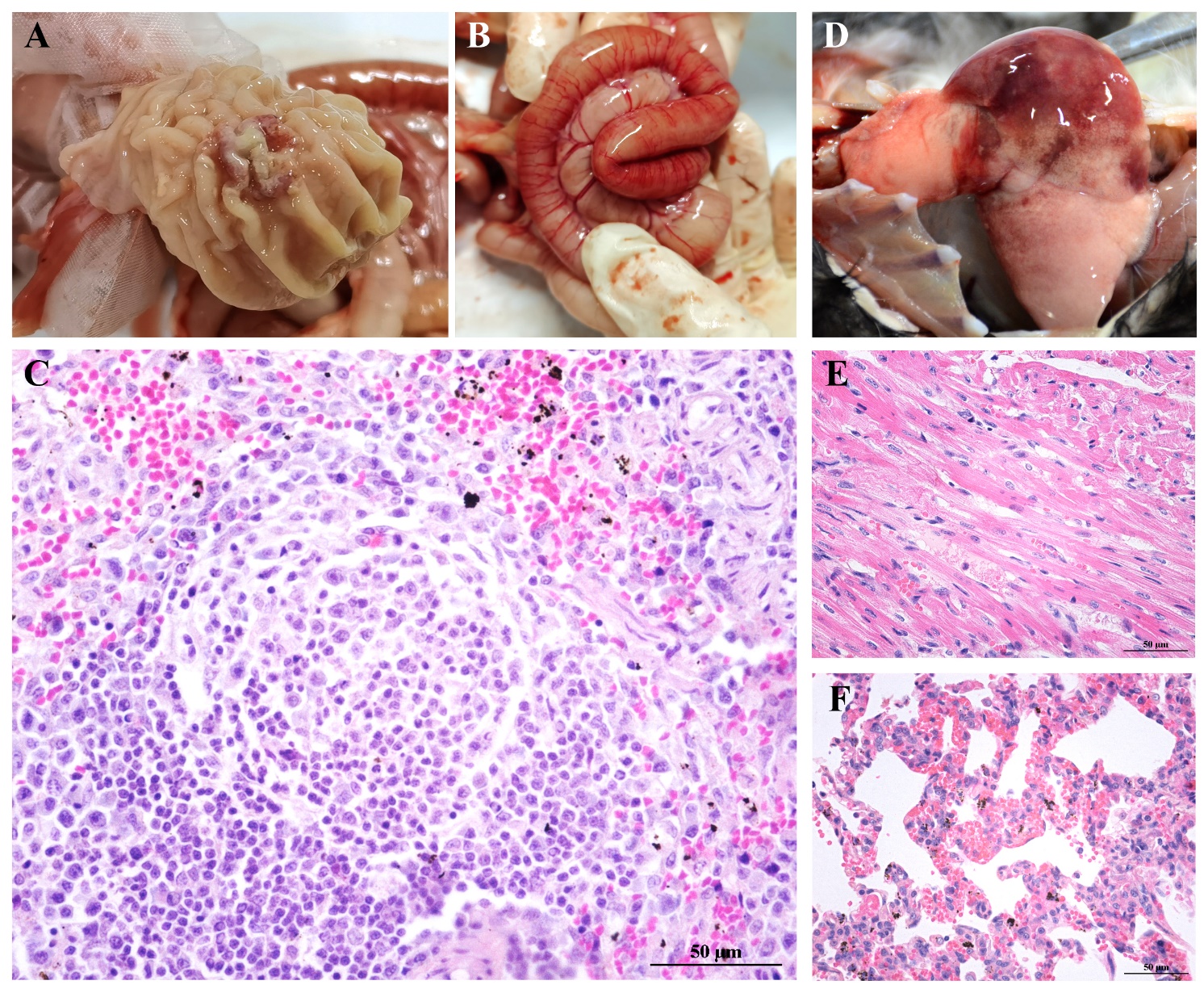


**Figure S2** The necropsy and histopathology of the challenged cats. (A) Necrosis in stomach. (B) Hemorrhagic enteritis. (C) Reduced lymphocytes in the white pulp. (D) Marked congestion in lung. (E) Degeneration and necrosis of the myocardium. (F) Congestion in the alveolar walls.


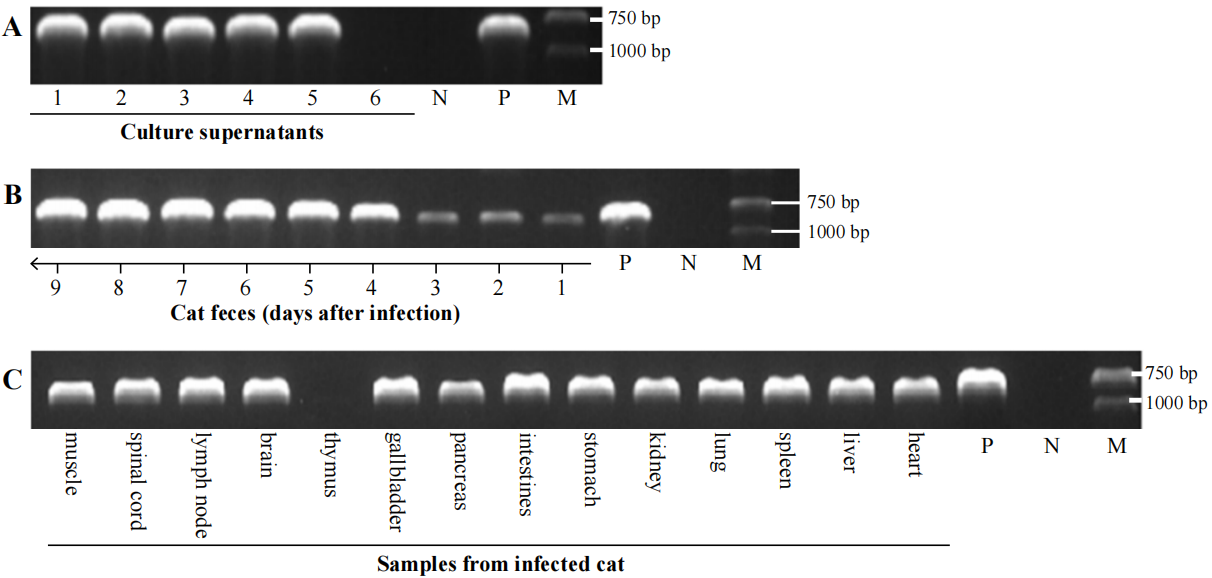


**Figure S3** The PCR results. (A) The PCR results of cell culture supernatant. Lane 6 was cells control group and Lanes 1- 5 were positive samples of F81 inoculated with virus and different blindly passages. (B) The PCR results of the cat feces. (C) The PCR results of tissues from the pathogenicity test. M: 2000 bp DNA marker; N: negative controls; P: positive controls.


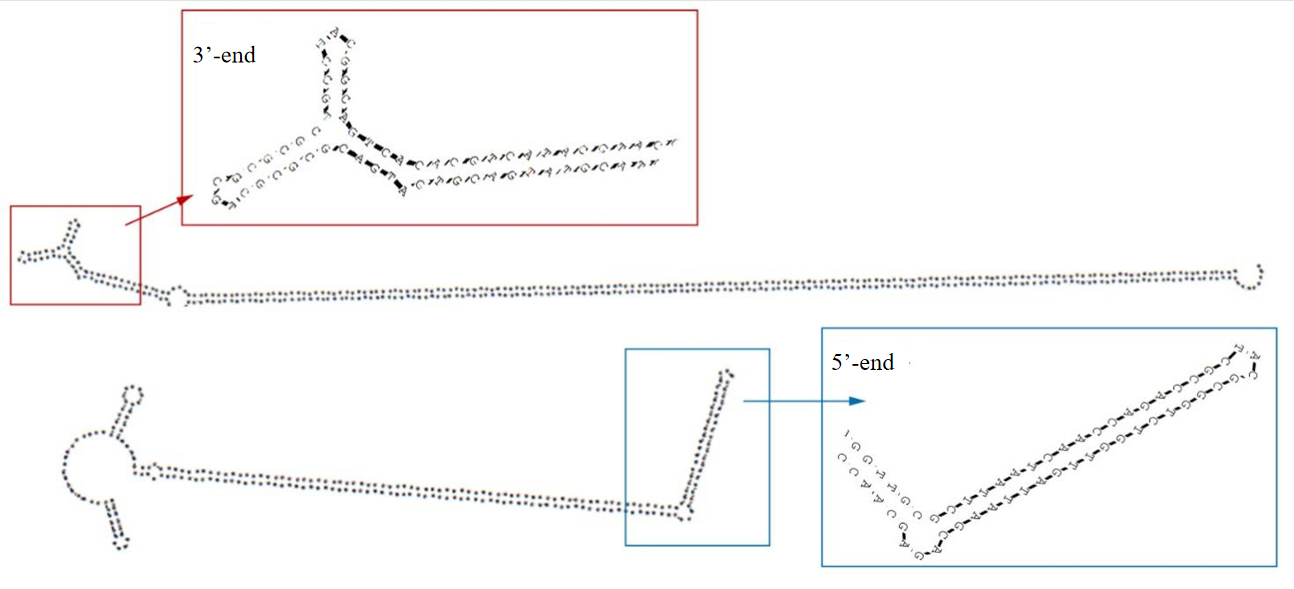


**Figure S4.** The palindromic sequence from 3' and 5' end of the genome assumed Y- shaped and U- shaped configurations respectively.

**Table S1** The results of daily PCR swab testing and PCR on tissues after necropsy.

|  | | Control group | | | Test group | | |
| --- | --- | --- | --- | --- | --- | --- | --- |
| PCR results | | C1 | C2 | C3 | T1 | T2 | T3 |
| Daily PCR swab testing | 0 d | - | - | - | - | - | - |
|  | 1 d | - | - | - | + | + | + |
|  | 2 d | - | - | - | + | + | + |
|  | 3 d | - | - | - | + | + | + |
|  | 4 d | - | - | - | + | + | + |
|  | 5 d | - | - | - | + | + | + |
|  | 6 d | - | - | - | + | × | + |
|  | 7 d | - | - | - | + | × | + |
|  | 8 d | - | - | - | + | × | + |
|  | 9 d | - | - | - | + | × | × |
| PCR on tissues after necropsy (heart, liver, spleen, lung, kidney, stomach, intestines, pancreas, gallbladder, brain, lymph node, spinal cord and muscle) | | - | - | - | + | + | + |
| PCR on tissues after necropsy (thymus) | | - | - | - | - | - | - |

“+”: PCR positive; “-”: PCR negative; “×”: No samples were collected

**Table S2.** All sequences (accession numbers and data) used in the phylogenetic analysis based on 36 whole genome sequences of CPPV-1.

| Accession numbers | Data |
| --- | --- |
| MZ712026 | FPV-am2020 (giant panda 2020 China) |
| MW145541.1 | CPPV-1 (wild fishing cat 2019 Thailand) |
| MH545963.1 | CPV-2a (dog 2018 India) |
| MF423123.1 | CPV-2b (coyote 2014 Canada) |
| MN840830.1 | CPV-2c (dog 2019 China) |
| MH476582.1 | CPV-new 2a (dog 2017 China) |
| MH476588.1 | CPV-new 2b (dog 2017 China) |
| KP280068.1 | FPV (domestic cat 2014 China) |
| KX685354.1 | FPV (tiger 2016 China) |
| KX900570.1 | FPV (jaguar 1986 China) |
| MF069445.1 | FPV (wild raccoon 2015 Canada) |
| MF069446.1 | FPV (wild raccoon 2010 Canada) |
| MF069447.1 | FPV (wild raccoon 2016 Canada) |
| MG764510.1 | FPV (Bengal tiger 1999 China) |
| MG764511.1 | FPV (lion 2015 China) |
| MG924893.1 | FPV (domestic cat 2016 China) |
| MH559110.1 | FPV (domestic cat 2018 India) |
| MN127779.1 | FPV (domestic cat 2018 Thailand) |
| MN127780.1 | FPV (domestic cat 2019 Thailand) |
| MN127781.1 | FPV (domestic cat 2019 Thailand) |
| MN451652.1 | FPV (Arctic Fox 1983 Finland) |
| MN862743.1 | FPV (wild American mink 2018 Canada) |
| MN862744.1 | FPV (wild American marten 2016 Canada) |
| MN862745.1 | FPV (wild American marten 2017 Canada) |
| MN862746.1 | FPV (wild American mink 2017 Canada) |
| MN862747.1 | FPV (wild American mink 2019 Canada) |
| MN862748.1 | FPV (wild North American river otter 2019 China) |
| MN862749.1 | FPV (wild North American river otter 2019 Canada) |
| MN908257.1 | FPV (South China tiger 2019 China) |
| MT614366.1 | FPV (domestic cat 2019 China) |
| MW650831.1 | FPV (domestic cat 2020 China) |
| MW811187.1 | FPV (domestic cat 2020 China) |
| MZ357120.1 | FPV (domestic cat 2020 China) |
| MZ357122.1 | FPV (giant panda 2018 China) |
| KT899745.1 | MEV (mink 2014 China) |
| KT899746.1 | MEV (mink 2010 China) |

**Table S3** All sequences (accession numbers and data) used in the phylogenetic analysis based on 235 *VP2* gene sequences of CPPV-1.

| Accession numbers | Data | Accession numbers | Data |
| --- | --- | --- | --- |
| MZ712026 | FPV-am2020 (giant panda 2020 China) | KU248461.1 | FPV (domestic cat 2006 Portugal) |
| EU698028.1 | BFPV (blue fox 2007 China) | KU248462.1 | FPV (domestic cat 2007 Portugal) |
| GQ857595.1 | BFPV (blue fox 2008 China) | KU248463.1 | FPV (domestic cat 2008 Portugal) |
| MN451652.1 | BFPV (Arctic fox 1983 Finland) | KU248464.1 | FPV (domestic cat 2014 Portugal) |
| MW145541.1 | CPPV-1 (wild fishing cat 2019 Thailand) | KX434461.1 | FPV (domestic cat 2015 Italy) |
| MW847155.1 | CPPV-1 (domestic cat 2011 Italy) | KX434462.1 | FPV (domestic cat 2015 Italy) |
| MW847156.1 | CPPV-1 (domestic cat 2011 Italy) | KX900570.1 | FPV (jaguar 1986 China) |
| MW847157.1 | CPPV-1 (domestic cat 2011 Italy) | M10824.1 | FPV (Crandell feline kidney cells 1985 U.S.) |
| MW847158.1 | CPPV-1 (domestic cat 2011 Italy) | M24004.1 | FPV (Crandell feline kidney cells 1988 U.S.) |
| MW847159.1 | CPPV-1 (domestic cat 2012 Italy) | M38246.1 | FPV (NA 1990 U.S.) |
| MW847160.1 | CPPV-1 (domestic cat 2012 Italy) | MF541128.1 | FPV (domestic cat 2016 China) |
| MW847161.1 | CPPV-1 (domestic cat 2012 Italy) | MG764511.1 | FPV (lion 2015 China) |
| MW847162.1 | CPPV-1 (domestic cat 2013 Italy) | MG924893.1 | FPV (domestic cat 2016 China) |
| MW847163.1 | CPPV-1 (domestic cat 2013 Italy) | MH165481.1 | FPV (domestic cat 2015 China) |
| MW847164.1 | CPPV-1 (domestic cat 2016 Italy) | MH165482.1 | FPV (domestic cat 2014 China) |
| MW847166.1 | CPPV-1 (domestic cat 2016 Italy) | MH559110.1 | FPV (domestic cat 2018 India) |
| MW847167.1 | CPPV-1 (domestic cat 2016 Italy) | MK266795.1 | FPV (domestic cat 2017 China) |
| MW847168.1 | CPPV-1 (domestic cat 2016 Italy) | MK357738.1 | FPV (st. huberthound 2017 Vietnam) |
| MW847169.1 | CPPV-1 (domestic cat 2016 Italy) | MN127779.1 | FPV (domestic cat 2018 Thailand) |
| MW847170.1 | CPPV-1 (domestic cat 2016 Italy) | MN127780.1 | FPV (domestic cat 2019 Thailand) |
| MW847171.1 | CPPV-1 (domestic cat 2016 Italy) | MN127781.1 | FPV (domestic cat 2019 Thailand) |
| MW847172.1 | CPPV-1 (domestic cat 2016 Italy) | MN400978.1 | FPV (domestic cat 2017 South Korea) |
| MW847173.1 | CPPV-1 (domestic cat 2016 Italy) | MN400979.1 | FPV (domestic cat 2017 South Korea) |
| MW847174.1 | CPPV-1 (domestic cat 2016 Italy) | MN400980.1 | FPV (domestic cat 2017 South Korea) |
| MW847175.1 | CPPV-1 (domestic cat 2016 Italy) | MN419003.1 | FPV (domestic cat 2019 China) |
| MW847176.1 | CPPV-1 (domestic cat 2016 Italy) | MN862748.1 | FPV (wild North American river otter 2019 China) |
| MW847177.1 | CPPV-1 (domestic cat 2016 Italy) | MN862749.1 | FPV (wild North American river otter 2019 Canada) |
| MW847178.1 | CPPV-1 (domestic cat 2017 Italy) | MT078767.1 | FPV (domestic cat 2019 India) |
| MW847179.1 | CPPV-1 (domestic cat 2017 Italy) | MT078768.1 | FPV (domestic cat 2019 India) |
| MW847180.1 | CPPV-1 (domestic cat 2017 Italy) | MT078769.1 | FPV (domestic cat 2019 India) |
| MW847181.1 | CPPV-1 (domestic cat 2017 Italy) | MT078770.1 | FPV (domestic cat 2019 India) |
| MW847182.1 | CPPV-1 (domestic cat 2017 Italy) | MT078771.1 | FPV (domestic cat 2019 India) |
| MW847183.1 | CPPV-1 (domestic cat 2017 Italy) | MT274377.1 | FPV (wild red fox 2017 Italy) |
| MW847184.1 | CPPV-1 (domestic cat 2017 Italy) | MT274378.1 | FPV (wild badger 2019 Italy) |
| MW847185.1 | CPPV-1 (domestic cat 2017 Italy) | MT614366.1 | FPV (domestic cat 2019 China) |
| MW847186.1 | CPPV-1 (domestic cat 2017 Italy) | MT857268.1 | FPV (domestic cat 2018 Vietnam) |
| MW847187.1 | CPPV-1 (domestic cat 2017 Italy) | MT857273.1 | FPV (domestic cat 2019 Vietnam) |
| MW847188.1 | CPPV-1 (domestic cat 2017 Italy) | MT857278.1 | FPV (domestic cat 2019 Vietnam) |
| MW847189.1 | CPPV-1 (domestic cat 2017 Italy) | MT892650.1 | FPV (domestic cat 2019 China) |
| MW847190.1 | CPPV-1 (domestic cat 2017 Italy) | MW017627.1 | FPV (domestic cat 2020 China) |
| MW847191.1 | CPPV-1 (domestic cat 2017 Italy) | MW650831.1 | FPV (domestic cat 2020 China) |
| MW847192.1 | CPPV-1 (domestic cat 2017 Italy) | MW811187.1 | FPV (domestic cat 2020 China) |
| MW847193.1 | CPPV-1 (domestic cat 2017 Italy) | MZ357120.1 | FPV (domestic cat 2020 China) |
| MW847194.1 | CPPV-1 (domestic cat 2017 Italy) | MZ357122.1 | FPV (giant panda 2018 China) |
| MW847195.1 | CPPV-1 (domestic cat 2017 Italy) | MZ836347.1 | FPV (domestic cat 2019 China) |
| MW847196.1 | CPPV-1 (domestic cat 2018 Italy) | MZ836350.1 | FPV (domestic cat 2020 China) |
| MW847197.1 | CPPV-1 (domestic cat 2018 Italy) | MZ836351.1 | FPV (domestic cat 2020 China) |
| MW847198.1 | CPPV-1 (domestic cat 2018 Italy) | MZ836352.1 | FPV (domestic cat 2020 China) |
| MW847199.1 | CPPV-1 (domestic cat 2018 Italy) | MZ836353.1 | FPV (domestic cat 2020 China) |
| MW847201.1 | CPPV-1 (domestic cat 2018 Italy) | MZ836354.1 | FPV (domestic cat 2020 China) |
| MW847202.1 | CPPV-1 (domestic cat 2018 Italy) | MZ836355.1 | FPV (domestic cat 2020 China) |
| MW847203.1 | CPPV-1 (domestic cat 2019 Italy) | MZ836356.1 | FPV (domestic cat 2020 China) |
| MW847204.1 | CPPV-1 (domestic cat 2019 Italy) | MZ836357.1 | FPV (domestic cat 2020 China) |
| MW847205.1 | CPPV-1 (domestic cat 2019 Italy) | MZ836358.1 | FPV (domestic cat 2020 China) |
| MW847206.1 | CPPV-1 (domestic cat 2019 Italy) | MZ836359.1 | FPV (domestic cat 2020 China) |
| MW847207.1 | CPPV-1 (domestic cat 2019 Italy) | MZ836360.1 | FPV (domestic cat 2020 China) |
| MW847208.1 | CPPV-1 (domestic cat 2018 Italy) | MZ836361.1 | FPV (domestic cat 2020 China) |
| MW847209.1 | CPPV-1 (domestic cat 2019 Italy) | MZ836362.1 | FPV (domestic cat 2020 China) |
| M38245.1 | CPV-2 (1978 U.S.) | MZ836363.1 | FPV (domestic cat 2020 China) |
| GU569948.1 | CPV-2a (1986 China) | MZ836364.1 | FPV (domestic cat 2020 China) |
| M24003.1 | CPV-2a (1984 U.S.) | MZ836365.1 | FPV (domestic cat 2020 China) |
| MH545963.1 | CPV-2a (2018 India) | MZ836366.1 | FPV (domestic cat 2020 China) |
| M74849.1 | CPV-2b (1984 U.S.) | MZ836367.1 | FPV (domestic cat 2020 China) |
| MF423123.1 | CPV-2b (wild 2014 Canada) | MZ836368.1 | FPV (domestic cat 2020 China) |
| FJ005196.1 | CPV-2c (1997 Germany) | MZ836369.1 | FPV (domestic cat 2020 China) |
| KF482468.1 | CPV-2c (2009 China) | MZ836370.1 | FPV (domestic cat 2020 China) |
| MN840830.1 | CPV-2c (wild 2019 China) | MZ836371.1 | FPV (domestic cat 2020 China) |
| FJ435347.1 | CPV-new 2a (2008 China) | MZ836373.1 | FPV (domestic cat 2020 China) |
| MH476582.1 | CPV-new 2a (2017 China) | MZ836374.1 | FPV (domestic cat 2020 China) |
| AY869724.1 | CPV-new 2b (2004 China) | MZ836375.1 | FPV (domestic cat 2020 China) |
| MH476588.1 | CPV-new 2b (2017 China) | MZ836376.1 | FPV (domestic cat 2020 China) |
| AB054227.1 | FPV (domestic cat 2000 Vietnam) | MZ836377.1 | FPV (domestic cat 2020 China) |
| AF015223.1 | FPV (NA 1997 China) | MZ836378.1 | FPV (domestic cat 2019 China) |
| D88286.1 | FPV (domestic cat 1990 Japan) | U22187.1 | FPV (wild cat 1990 U.S.) |
| EF418569.1 | FPV (lion 2006 Portugal) | U22188.1 | FPV (wild cat 1993 Germany) |
| EF988660.1 | FPV (domestic cat 2007 China) | U22189.1 | FPV (wild cat 1964 U.S.) |
| EU018142.1 | FPV (domestic cat 2007 Argentina) | X55115.1 | FPV (domestic cat 1970 Australia) |
| EU018143.1 | FPV (domestic cat 2007 Argentina) | D00765.1 | MEV (mink 1978 Japan) |
| EU018144.1 | FPV (domestic cat 2007 Argentina) | FJ712218.1 | MEV (mink 2008 China) |
| EU018145.1 | FPV (domestic cat 2007 Argentina) | FJ712219.1 | MEV (mink 2008 China) |
| EU145593.1 | FPV (wild Asian palm civet 2007 Hungary) | FJ712220.1 | MEV (mink 2008 China) |
| EU221278.1 | FPV (domestic cat 2005 Portugal) | FJ712221.1 | MEV (mink 2008 China) |
| EU221279.1 | FPV (domestic cat 2005 Portugal) | KT899745.1 | MEV (mink 2014 China) |
| EU221280.1 | FPV (domestic cat 2006 Portugal) | KT899746.1 | MEV (mink 2010 China) |
| EU221281.1 | FPV (domestic cat 2006 Portugal) | KY094114.1 | MEV (mink 2015 China) |
| EU498680.1 | FPV (vaccine Purevax RCP Merial) | KY094117.1 | MEV (mink 2016 China) |
| EU498681.1 | FPV (vaccine Felocell CVR Pfizer) | M24001.1 | MEV (mink 1967 Canada) |
| EU498692.1 | FPV (domestic cat 2004 Italy) | MN862743.1 | MEV (wild American mink 2018 Canada) |
| EU659112.1 | FPV (domestic cat 1964 U.S.) | MN862744.1 | MEV (wild American marten 2016 Canada) |
| EU659113.1 | FPV (mountain lion 1989 U.S.) | MN862745.1 | MEV (wild American marten 2017 Canada) |
| EU659114.1 | FPV (mountain lion 1989 U.S.) | MN862746.1 | MEV (wild American mink 2017 Canada) |
| EU659115.1 | FPV (domestic cat 2006 U.S.) | MN862747.1 | MEV (wild American mink 2019 Canada) |
| FJ231389.1 | FPV (monkey 2008 China) | MH213135.1 | RDPV (raccoon dog 2018 South Korea) |
| FJ440711.1 | FPV (domestic cat 2007 Argentina) | MH643886.1 | RDPV (wild raccoon dog 2016 South Korea) |
| FJ440712.1 | FPV (domestic cat 2007 Argentina) | JN867593.1 | RPV (wild raccoon 2010 U.S.) |
| FJ440713.1 | FPV (domestic cat 2007 Argentina) | JN867594.1 | RPV (wild raccoon 1990 U.S.) |
| FJ440714.1 | FPV (domestic cat 2007 Argentina) | JN867595.1 | RPV (wild raccoon 1978 U.S.) |
| HQ184189.1 | FPV (domestic cat 2008 South Korea) | JN867596.1 | RPV (wild raccoon 1978 U.S.) |
| HQ184190.1 | FPV (domestic cat 2008 South Korea) | KJ813894.1 | RPV (wild raccoon 2012 U.S.) |
| HQ184191.1 | FPV (domestic cat 2008 South Korea) | KJ813895.1 | RPV (wild raccoon 2012 U.S.) |
| JF422105.2 | FPV (wild Egyptian mongoose 2009 Portugal) | KM624023.1 | RPV (wild raccoon 1978 U.S.) |
| KJ813893.1 | FPV (wild bobcat 2013 U.S.) | M24005.1 | RPV (wild raccoon 1988 U.S.) |
| KP280068.1 | FPV (domestic cat 2014 China) | MF069445.1 | RPV (wild raccoon 2015 Canada) |
| KP769859.1 | FPV (domestic cat 2013 Belgium) | MF069446.1 | RPV (wild raccoon 2010 Canada) |
| KT240128.1 | FPV (domestic cat 2006 Portugal) | MF069447.1 | RPV (wild raccoon 2016 Canada) |
| KT240129.1 | FPV (domestic cat 2007 Portugal) | MH669800.1 | RPV (wild raccoon 1978 U.S.) |
| KT240130.1 | FPV (domestic cat 2008 Portugal) | AY955826.1 | TPV (tiger 2005 China) |
| KT240131.1 | FPV (domestic cat 2008 Portugal) | DQ099430.1 | TPV (tiger 2005 China) |
| KT240132.1 | FPV (domestic cat 2012 Portugal) | EF418568.1 | TPV (Bengal tiger 2006 Portugal) |
| KT240133.1 | FPV (domestic cat 2013 Portugal) | EU697383.1 | TPV (tiger 2007 China) |
| KT240134.1 | FPV (domestic cat 2013 Portugal) | EU697384.1 | TPV (tiger 2007 China) |
| KT240135.1 | FPV (domestic cat 2014 Portugal) | EU697386.1 | TPV (tiger 2007 China) |
| KT240136.1 | FPV (domestic cat 2014 Portugal) | EU697387.1 | TPV (tiger 2007 China) |
| KU248456.1 | FPV (domestic cat 2006 Portugal) | FJ405225.1 | TPV (tiger 2008 China) |
| KU248457.1 | FPV (domestic cat 2006 Portugal) | KX685354.1 | TPV (tiger 2016 China) |
| KU248458.1 | FPV (domestic cat 2006 Portugal) | MG764510.1 | TPV (Bengal tiger 1999 China) |
| KU248459.1 | FPV (domestic cat 2006 Portugal) | MN908257.1 | TPV (South China tiger 2019 China) |
| KU248460.1 | FPV (domestic cat 2006 Portugal) |  |  |
